# Characterization of a knock-in mouse model of the homozygous p.V37I variant in *Gjb2*

**DOI:** 10.1038/srep33279

**Published:** 2016-09-13

**Authors:** Ying Chen, Lingxiang Hu, Xueling Wang, Changling Sun, Xin Lin, Lei li, Ling Mei, Zhiwu Huang, Tao Yang, Hao Wu

**Affiliations:** 1 Department of Otorhinolaryngology-Head and Neck Surgery, Shanghai Ninth People’s Hospital, Shanghai Jiaotong University School of Medicine, Shanghai, China; 2 Ear Institute, Shanghai Jiaotong University, Shanghai, China; 3 Shanghai Key Laboratory of Translational Medicine on Ear and Nose Diseases, Shanghai, China; 4 Department of Otorhinolaryngology-Head and Neck Surgery, Xinhua Hospital, Shanghai Jiaotong University School of Medicine, Shanghai, China

## Abstract

The homozygous p.V37I variant in *GJB2* is prevalent in East and Southeast Asians and may lead to mild-to-moderate hearing loss with reduced penetrance. To investigate the pathogenic mechanism underlying this variant, we generated a knock-in mouse model of homozygous p.V37I by an embryonic stem cell gene targeting method. Auditory brainstem response test showed that the knock-in mice developed progressive, mild-to-moderate hearing loss over the first 4–9 months. Overall no significant developmental and morphological abnormality was observed in the knock-in mouse cochlea, while confocal immunostaining and electron microscopic scanning revealed minor loss of the outer hair cells. Gene expression microarray analysis identified 105 up-regulated and 43 down-regulated genes in P5 knock-in mouse cochleae (*P* < 0.05 adjusted by the Benjamini & Hochberg method), among which four top candidate genes with the highest fold-changes or implication to deafness *Fcer1g, Nnmt* and *Lars2* and *Cuedc1* were verified by quantitative real-time PCR. Our study demonstrated that the homozygous p.V37I knock-in mouse modeled the hearing phenotype of the human patients and can serve as a useful animal model for further studies. The differentially expressed genes identified in this study may shed new insights into the understanding of the pathogenic mechanism and the phenotypic modification of homozygous p.V37I.

Hearing loss is the most common sensory disorder in humans, affecting approximately 2–3% of children^1^. It was estimated that at least 50% to 60% of childhood hearing loss is attributable to genetic causes. Among them, recessive mutations in *GJB2* are the most frequent, accounting for 20–30% of cases in many countries and ethnic groups^2^,^3^.

*GJB2* encodes the gap junction subunit protein connexin 26 (Cx26). It comprises 226 amino acids with 4 transmembrane domains. Together with over 20 other connexin subunit proteins, Cx26 forms hexameric hemichannels (connexons) on opposite cellular membranes, allowing intercellular communication between adjacent cells. In cochlea, *Gjb2* is expressed in the non-sensory cells and structures including the supporting cells, the stria vascularis, the spiral ligament and the spiral limbus^4^,^5^. The function of *Gjb2* was implicated in many auditory processes including potassium recycling, nutrient and energy supply, generation of endocochlear potential and maintenance of the endolymphatic homeostasis in the inner ear^6^.

To date, over 300 mutations in *GJB2* (The Human Gene Mutation Database, http://www.hgmd.cf.ac.uk/ac/) have been reported causing recessive non-syndromic deafness DFNB1 (MIM# 220290). The hearing loss associated with DFNB1 was highly variable, ranging from birth to adulthood in age of onsets and from mild to profound in degree of severity. Based on the deleterious impact on the protein structure, most of the *GJB2* mutations can be categorized into truncating mutations (*e.g.* non-sense mutations, frameshifting indels, splicing site mutations) that often lead to null alleles of *GJB2* and non-truncating mutations (*e.g.* missense mutations, non-frameshifting indels) that only affecting single or multiple amino acids. Genotype-phenotype correlation studies showed that the degree of hearing loss associated with non-truncating mutations in *GJB2* was significantly less severe than that with truncating mutations^7^. One such example, the p.V37I variant in *GJB2*, is of our particular interest as it is prevalent in East and Southeast Asians, with allele frequencies of 0.062 in Chinese Hans, 0.043 in Thai, 0.01 in Japanese and 0.006 in Korean^8^,^9^,^10^,^11^. *In vitro* studies showed that Cx26 with truncating mutations cannot target to the cell membrane and has severely deteriorated gap junction activity^12^. On the contrary, the p.V37I mutant Cx26 had normal cellular expression pattern at the plasma membrane and moderately impaired oligomerization and channel activity^10^,^13^,^14^. Consistently, the homozygous p.V37I variant of *GJB2* has been shown to be strongly associated with mild-to-moderate hearing loss. This variant, however, has reduced penetrance which was estimated to be 17%^8^.

The pathogenic mechanism underlying the milder degree of phenotype and incomplete penetrance of the homozygous p.V37I variant remained unclear. So far, animal models of DFNB1 have only been established for conditional null alleles of *Gjb2*, which exhibits a rather early and severe hearing phenotype. Targeted ablation of *Gjb2* in mouse inner ear resulted in elevation of the hearing thresholds by 40–50 dB at postnatal day 21 (P21), stalled postnatal development of the organ of Corti as the tunnel of Corti and the Nuel’s space were never opened, and massive cell death of the sensory epithelium in the middle and basal turns of cochlea^15^,^16^. In this study, we aimed to generate a knock-in mouse model for the homozygous p.V37I variant in *Gjb2*, which may help to better understand why homozygous p.V37I is associated with milder degree of hearing loss than truncating mutations and what factors may contribute to the incomplete penetrance of homozygous p.V37I.

## Results

### The homozygous p.V37I knock-in mice have progressive, mild-to-moderate hearing loss

**Figure 1:**
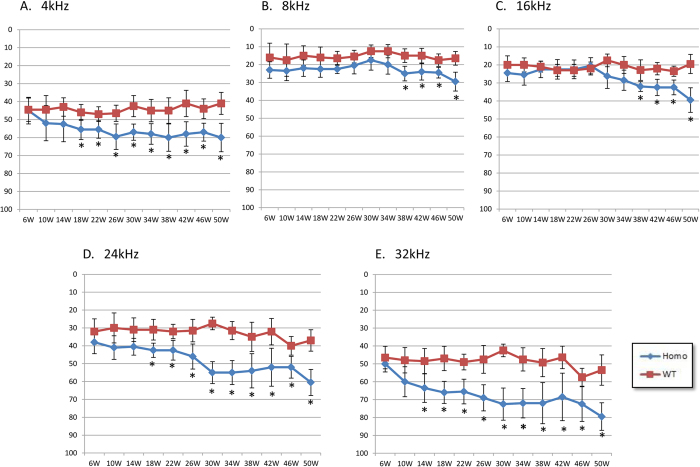
ABR thresholds of the homozygous p.V37I knock-in (KI) and wild-type (WT) mice. (**A–E**) The averaged ABR thresholds of the p.V37I homozygous (Homo) and wild-type (WT) mice (n = 10 each) between age 6 weeks and 50 weeks at 4, 8, 16, 24 and 32 kHz. Asterisks indicate the statistical significance with *P*-values of 0.05 or lower.

Hearing thresholds of the homozygous p.V37I knock-in and wild-type mice were consecutively monitored at frequencies of 4, 8, 16, 24 and 36 kHz between age 6 and 50 weeks by auditory brainstem response (ABR) tests (Fig. 1). Elevated hearing thresholds were observed in all knock-in mice at all frequencies, while the degrees and onsets of the hearing loss were moderately variable. Since 18 weeks, the knock-in mice showed statistically significant (*P* < 0.05) increase of the hearing thresholds at 4, 24 and 32 kHz. This increase was statistically significant since 38 weeks at 8 and 16 kHz. Our results showed that the homozygous p.V37I knock-in mice modeled the progressive, mild-to-moderate hearing loss in human patients.

### The homozygous p.V37I knock-in mice have normal cochlear morphology but minor loss of outer hair cells

**Figure 2:**
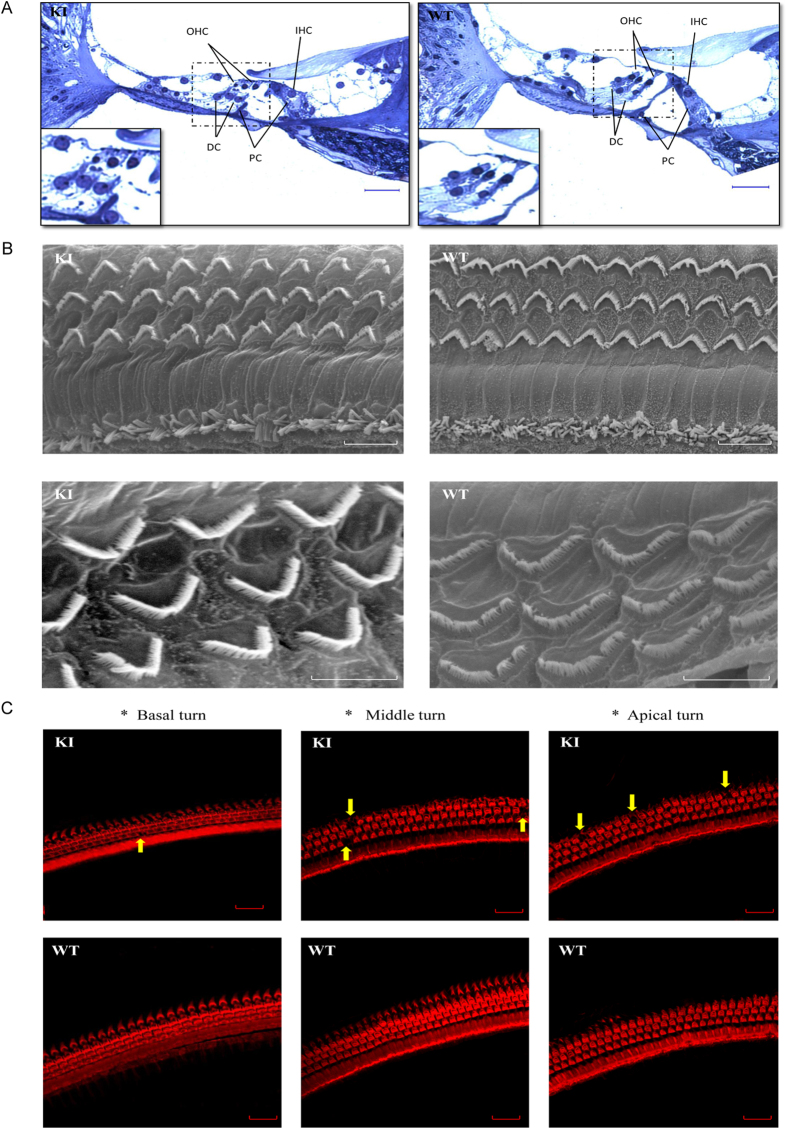
Cochlea morphology of the homozygous p.V37I knock-in (KI) and wild-type (WT) mice at age one year. (**A**) Semithin histology sections showing the normal gross morphology of the KI mouse cochleae. Scale bars: 100 μm. (**B**) Scanning electron microscopy showing normal cellular structure and arrangement of the hair cell bundles of the KI mice. Scale bars: 10 μm (top) and 5 μm (bottom). (**C**) Confocal immunofluorescence microscopy showing minor loss of the outer hair cells (arrows) at the apical, middle and basal turns of the KI mouse cochleae. F-actin was stained with rhodamine phalloidin (1:100) to identify the hair cells. Scale bars: 20 μm.

The gross cochlear development and morphology in the homozygous p.V37I knock-in mice appeared normal (Fig. 2A). Scanning electron microscopy (SEM) showed that the cellular structure and arrangement of the hair cell bundles remained intact (Fig. 2B). Both SEM and immunofluorescence microscopy, however, revealed minor loss of outer hair cells at age one year (approximately 1.3% at the apical turns and 3.8% at the basal and middle turns of the cochlea, Fig. 2C and Supplementary Table S2).

### *Fcer1g, Nnmt, Cuedc1* and *Lars2* were differentially expressed in the homozygous p.V37I knock-in mouse cochleae

**Figure 3:**
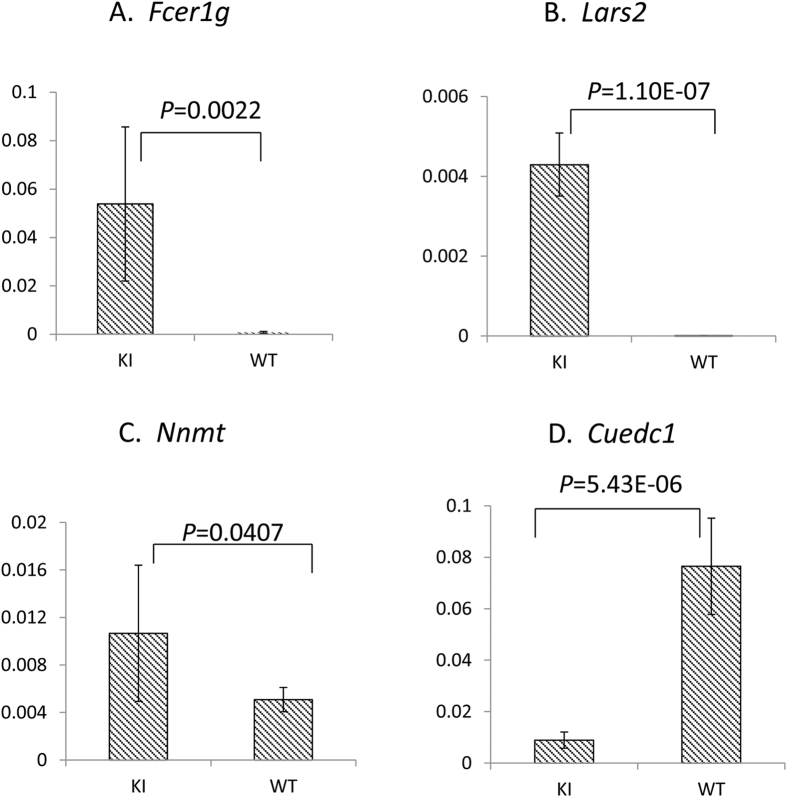
Differential expression of *Fcer1g, Lars2, Nnmt* and *Cuedc1* in the homozygous p.V37I knock-in (KI) mice. qRT-PCR was performed in inner ear cDNA samples of KI and WT mice (n = 6 each). Expression levels of *Fcer1g, Lars2, Nnmt* and *Cuedc1* were normalized to that of the endogenous *Gapdh* controls.

To investigate the early pathogenic pathway through which the homozygous p.V37I variant of *Gjb2* leads to eventual progressive hearing loss, we performed a gene expression microarray analysis in randomly chosen P5 knock-in and wild-type mouse cochleae. A total of 105 up-regulated and 43 down-regulated genes were identified in the knock-in mouse cochleae (*P* < 0.05 after the Benjamini & Hochberg correction for multiple testing^17^, Supplementary Table S1). The differential expression of six candidate genes were further validated by quantitative real-time PCR (qRT-PCR), which included the top up-regulated (*Fcer1g, Nnmt*) and down-regulated genes (*Cuedc1, Slc30a10* and *Gpr126*) with the highest fold changes as well as a gene (*Lars2*) previously implicated in syndromic deafness Perrault syndrome. The up-regulation of *Fcer1g, Lars2*, *Nnmt* and down-regulation of *Cuedc1* were confirmed by qRT-PCR (*P* = 0.002, 1.1 × 10^−7^, 0.041 and 5.4 × 10^−6^, respectively), while the down-regulation of *Slc30a10* and *Gpr126* were not (*P* > 0.05, Fig. 3).

## Discussion

The p.V37I variant in *GJB2* has by far the highest allele frequency (0.062 in Chinese Hans) among all reported variants associated with hearing loss. It was estimated that over 4 million people among East and Southeast Asians will be homozygous for p.V37I and genetically susceptible for mild-to-moderate hearing loss^8^. Better understanding of the pathogenic mechanism and the genotype-phenotype correlation for this variant, therefore, may broadly facilitate clinical intervention and genetic counseling among the homozygous p.V37I carriers.

In this study, we generated a knock-in mouse model for the homozygous p.V37I variant in *Gjb2*. In comparison with the *Gjb2* knock-out mice, whose hearing thresholds were elevated by 40–50 dB at P21^16^, the homozygous p.V37I knock-in mice had much milder degree of hearing loss with later onset and slower progression course (Fig. 1), which modeled its representative hearing phenotype in human patients. Consistent with the milder phenotype, the homozygous p.V37I knock-in mice have overall normal cochlear development and morphology and only minor outer hair cell loss at 50 weeks (Fig. 2). Though the minor loss of outer hair cells (<4%) is less likely to be solely responsible for the elevated hearing thresholds observed in the knock-in mice, it may reflect the underlying pathophysiological changes of the knock-in mice that were not apparent at the morphological level.

Though late-onset hearing loss was associated with homozygous p.V37I in both human patients and the mouse model, many p.V37I homozygous patients with late-onset hearing loss exhibited subclinical hearing loss (<35 dB nHL at diagnosis but failed the initial otoacoustic emission hearing screening) at birth, implying that the pathophysiological process can be accumulative and begin in much earlier age^18^. Consistently, deletion of Cx26 expression at P5 can also lead to late-onset hearing loss in mice^19^. To explore the early pathogenic pathway through which the homozygous p.V37I variant of *Gjb2* leads to eventual progressive hearing loss, we performed an inner ear gene expression microarray analysis for the P5 knock-in and wild-type mice. A total of 148 genes showed significant up-regulation (n = 105) or down-regulation (n = 43) in the knock-in mice (Supplementary Table S1), which provided a candidate list for genes potentially involved in the pathogenic process. The differential expression of four candidate genes, *Fcer1g, Nnmt, Lars2* and *Cuedc1*, was further confirmed by qRT-PCR (Fig. 3). *Fcer1g*, *Nnmt* and *Cuedc1* were three top candidate genes with the highest fold changes in the microarray analysis. *Fcer1g* encodes the gamma chain of the high-affinity IgE receptor that is involved in allergic reactions^20^. *Nnmt* encodes Nicotinamide N-methyltransferase that is involved in homocysteine synthesis^21^. *Cuedc1* encodes a CUE domain protein with undefined function. Our literature search did not return any link between those three genes and the hearing function. *Lars2,* on the other hand, was an interesting candidate as mutations in this gene may lead to premature ovarian failure and hearing loss in Perrault syndrome (MIM 604544)^22^. *Lars2* encodes mitochondrial leucyl-tRNA synthetase. Mutations in many genes encoding this type of aminoacyl-tRNA synthetases, such as *HARS2*, *KARS*, *NARS* and *LARS2*, have been reported to be associated with non-syndromic or syndromic hearing loss^22^,^23^,^24^,^25^, suggesting the importance of those genes in the inner ear function. We speculated that the decreased expression level of Lars2 triggered by the p.V37I homozygous variant of *Gjb2* may contribute to the hearing loss in the knock-in mice.

In summary, our study generated a homozygous p.V37I knock-in mouse model that modeled the representative hearing phenotype in human patients and provided a valuable tool for further functional studies of this variant. One intriguing question about the homozygous p.V37I variant in *GJB2* is to discover the factors that may contribute to its incomplete penetrance, which was estimated to be 17%^8^. In future studies, it will be interesting to examine the impact of various environmental conditions, such as noise and aminoglycoside exposure, on the severity and onset of the hearing loss in the knock-in mice. On the other side, further genetic and molecular studies of the knock-in mice, like the in-depth analysis of the differentially expressed genes following the current study, may identify the genetic modifiers influencing the hearing phenotype.

## Methods and Materials

### Generation of the p.V37I knock-in mouse model

The p.V37I knock-in mouse model was generated by embryonic stem (ES) cell gene targeting as previously described^26^. Briefly, Fragments from bacterial artificial chromosome (BAC) containing the mouse *Gjb2* genomic region (bMQ-247e22, Source Bioscience, Nottingham, UK) were assembled into the targeting vector (pBR322-MK, modified by Shanghai Biomodel Organism Science & Technology Development Co., Shanghai, China). A *loxP-neo* cassette was inserted into the intron 1 of *Gjb2* in this vector (Supplementary Figure 1). The p.V37I (c.109G > A) mutation was introduced into the vector by site-directed mutagenesis with the QuikChange Site-Directed Mutagenesis kit (Stratagene, La Jolla, CA, USA). Gene targeting was performed by electrophoresis using embryonic stem cells EL-350 (Given by Wellcome Trust Sanger Institute, UK) at 1.5 × 10^7^/ml. The positive clones were screened by PCR analysis of the genomic DNA followed by sequencing analysis. After germline transmission of the properly targeted ES cell clone, the knock-in mice were obtained by crossing with the 129T2/SvEmsJ wild-type strain. The genotypes of the p.V37I mutant mice were confirmed by sequencing of the mouse tail genomic DNA. The experimental protocol was approved by the Ethics Committee of Xinhua Hospital, Shanghai Jiaotong University School of Medicine and performed in accordance with the guideline for animal experiments of Xinhua Hospital, Shanghai Jiaotong University School of Medicine.

### ABR tests

ABR tests were performed in wild-type (n = 10) and homozygous p.V37I (n = 10) mice every four weeks between age 6 weeks and 50 weeks as previously described 27. Briefly, mice were anesthetized with ketamine (90 mg/kg) and xylazine (10 mg/kg) by intraperitoneal injection. Tone bursts of various frequencies ranging from 4 kHz to 32 kHz (10 ms in duration and a rise–fall time of 0.5 ms) were generated by the BioSigRZ workstation (Tucker–Davis Technologies, Alachua, FL, USA). The ABR threshold was measured based on the presence of wave II in a series of repeatable ABR responses obtained at various sound intensities. Pure tone ABR threshold was measured at 4, 8, 16, 24 and 32 kHz. The genotypes of the mice were kept confidential to the tester at the time of the experiment.

### Cochlear histology analysis

Mouse cochleae were harvested and fixed with 2.5% glutaraldehyde for overnight at 4 degrees Celsius. After decalcification with 0.1 M EDTA for 14 days, the cochleae were fixed with 1% OsO4, dehydrated with ethanol of increasing grades, infiltrated with acetone and final resin, placed in capsules contained embedding medium and heated at 70 °C for 9 hours. Semithin sections of cochleae were cut in thickness of 5 μm, stained with methylaniline blue and observed under microscope DMI 3000 (Leica Microsystems, Wetzlar, Germany).

### SEM

Mouse cochleae were fixed with 2.5% glutaraldehyde for 2 hours, washed three times in PBS and decalcificated with 0.1 M EDTA for 2 hours. The lateral wall was carefully removed and the hair cells were exposed on the modiolus. The cochleae were then fixed with 1% OsO4, dehydrated with ethanol of increasing grades, immersed into the mixture of alcohol and iso-amylacetate followed by iso-amylacetate, dehydrated, coated with gold-palladium and observed under scanning electron microscopy TM-1000 (Philips, Sunnyvale, CA, USA).

### Immunofluorescence microscopy

Mouse cochleae were perfused and fixed with 4% paraformaldehyde for 4 hours at room temperature. After decalcification with 0.1 M EDTA for 14 days, the organs of Corti were microdissected under a microscope. F-actin was stained with rhodamine phalloidin (1:100) to identify the hair cells at the basal, middle and apical turns of the cochlea under a Leica TCS SPE confocal microscope (Leica Microsystems).

### Gene expression microarray analysis

Gene expression microarray analysis was performed in homozygous p.V37I knock-in and wild-type mice at P5 (n = 6 each). Cochleae without the vestibular part of the inner were harvested from the sacrificed mice and immediately immersed in the RNAlater RNA stabilization reagent (Qiagen, Shanghai, China). Total mRNA samples were extracted using the TRIzol reagent (Invitrogen, Carlsbad, CA, USA) and reverse-transcribed to cDNA using the PrimeScript™ RT Master kit (TAKARA, Kusatsu, Japan). Hybridization was performed using the Illumina MouseWG-6 v2 Expression Beadchip system (Illumina, San Diego, CA, USA), which contains 45200 probes per array product. Normalization algorithms were used to transfer sample signals to minimize the effects of variation arising from nonbiological factors. Analysis of gene expression profiles was completed by the Illumina BeadStudio module application (Illumina). A twofold or greater difference in the intensity of gene expression between the mutant and wild-type mice was sought. Up-regulated or down-regulated genes were cataloged. The *P* values were calculated by *t*-test with unpaired unequal variance (Welch) and adjusted for multiple testing by the Benjamini & Hochberg method^17^.

### qRT-PCR

The differential expression of *Fcer1g, Nnmt, Cuedc1, Slc30a10, Gpr126* and *Lars2* was further evaluated by SYBR green-based qRT-PCR in mouse inner ear cDNA samples of knock-in and wild-type mice (n = 6 each). qRT-PCR primers and probes of each gene were designed using ABI Primer Express Software v2.0 (Applied Biosystems, Foster City, CA, USA). qRT-PCR analysis of the cDNA samples was performed using the ABI Prism 7700 sequence detection system (Applied Biosystems) following the standard procedure. Data were analyzed using the ABI Prism 7500 SDS software (Applied Biosystems). Quantitative expression data of each specific target were obtained for each cDNA sample. Expression of *Gapdh* was used as endogenous normalization control. The comparative threshold of cycle (*C*_T_) method was used to determine any difference in target expression between the homozygous p.V37I knock-in and wild-type mice. All values were log_2_ transformed before statistical anaylsis.

### Statistics

Fisher’s exact test was used to compare the ABR thresholds of the knock-in and wild-type mice^28^. *P* values were presented as the result of the two-tailed analysis. For gene expression microarray analysis, *P* values were calculated by *t*-test with unpaired unequal variance (Welch) and adjusted for multiple testing by the Benjamini & Hochberg method^17^. Mann-Whitney test was used to compare the expression levels of the knock-in and wild-type mice by qRT-PCR^29^.

## Additional Information

**How to cite this article**: Chen, Y. *et al.* Characterization of a knock-in mouse model of the homozygous p.V37I variant in *Gjb2. Sci. Rep.* **6**, 33279; doi: 10.1038/srep33279 (2016).

## Supplementary Material

Supplementary Information
