# Supplementary material for: Characterization of a knock-in mouse model of the homozygous p.V37I variant in Gjb2
Source: Sci Rep. 2016 Sep 13;6:33279. doi: 10.1038/srep33279 (PMC5020688; doi:10.1038/srep33279)

**Characterization of a knock-in mouse model of the homozygous p.V37I variant in *Gjb2***

Ying Chen^1,2,3,4,§^, Lingxiang Hu^2,3,4,§^, Xueling Wang^2,3,4^, Changling Sun^2,3,4^, Xin Lin^2,3,4^, Lei li^2,3,4^, Ling Mei^2,3,4^, Zhiwu Huang^2,3,4^, Tao Yang^2,3,4^*****, Hao Wu^1,2,3,4^*****

^1^ Department of Otorhinolaryngology-Head and Neck Surgery, Shanghai Ninth People’s Hospital, Shanghai Jiaotong University School of Medicine, Shanghai, China

^2^ Ear Institute, Shanghai Jiaotong University, Shanghai, China

^3^Shanghai Key Laboratory of Translational Medicine on Ear and Nose Diseases, Shanghai, China.

^4^ Department of Otorhinolaryngology-Head and Neck Surgery, Xinhua Hospital, Shanghai Jiaotong University School of Medicine, Shanghai, China

^§^Those two authors contributed equally to this work

***Corresponding Authors:**

Hao Wu, Department of Otolaryngology-Head & Neck Surgery, Shanghai Ninth People’s Hospital, Shanghai Jiaotong University School of Medicine, Shanghai, China. Email: wuhao622@sina.cn, Tel: 86-21-55570010, Fax: +86-21-65152394

Tao Yang, Ear Institute, Shanghai Jiaotong University School of Medicine, Shanghai, China. Email: [yangtfxl@sina.com](mailto:yangtfxl@sina.com), Tel: 86-21-25078893, Fax: 86-21-65152394

**Supplementary Table 1. Differentially expressed genes in homozygous p.V37I knock-in mouse cochleae**

| Probe ID | Gene symbol | Fold change | FDR |
| --- | --- | --- | --- |
| ILMN_2748875 | *FCER1G* | 160.74 | 2.56E-04 |
| ILMN_2544305 | *NNMT* | 63.59 | 4.54E-03 |
| ILMN_2534207 | *LOC380706* | 44.06 | 2.66E-04 |
| ILMN_1230858 | *9430065F12RIK* | 39.73 | 2.80E-05 |
| ILMN_2692989 | *2810410P22RIK(AR15A)* | 36.30 | 1.74E-03 |
| ILMN_2549473 | *LOC667034(PNP2)* | 35.39 | 2.50E-05 |
| ILMN_2703061 | *2810408P10RIK* | 25.28 | 1.20E-05 |
| ILMN_1223381 | *PADI2* | 24.90 | 9.33E-04 |
| ILMN_2692986 | *2810410P22RIK* | 24.74 | 6.73E-03 |
| ILMN_2589318 | *POU6F1* | 22.79 | 1.18E-02 |
| ILMN_2898944 | *LRRC57* | 22.02 | 6.48E-03 |
| ILMN_2558387 | *KCNQ5* | 20.69 | 2.52E-03 |
| ILMN_1226174 | *ENTPD4* | 19.91 | 7.14E-07 |
| ILMN_1249638 | *RBBP4* | 18.45 | 5.90E-03 |
| ILMN_1249943 | *6430530L21RIK* | 17.06 | 3.10E-04 |
| ILMN_1251419 | *MELA* | 17.04 | 9.55E-03 |
| ILMN_2597332 | *1700123O20RIK* | 15.52 | 1.57E-02 |
| ILMN_1245266 | *A130084F23RIK* | 14.34 | 3.44E-05 |
| ILMN_2768053 | *SUPT16H* | 13.64 | 1.46E-04 |
| ILMN_2572307 | *PTPRG* | 13.39 | 1.59E-03 |
| ILMN_3090731 | *PKIG* | 13.32 | 1.32E-02 |
| ILMN_2654377 | *RYA3* | 13.18 | 1.49E-02 |
| ILMN_2749976 | *BAT4* | 11.40 | 1.65E-02 |
| ILMN_1218923 | *LOC100041932* | 11.24 | 2.07E-02 |
| ILMN_2554110 | *ENTPD4* | 10.47 | 2.69E-02 |
| ILMN_1221341 | *LOC100047226* | 10.34 | 2.89E-02 |
| ILMN_1233004 | *D930049F02RIK* | 9.90 | 1.02E-02 |
| ILMN_2503166 | *C330011F01RIK* | 9.49 | 3.75E-02 |
| ILMN_3162426 | *RNASE12* | 9.28 | 7.99E-03 |
| ILMN_1217271 | *C130067A03RIK* | 9.12 | 1.17E-02 |
| ILMN_2632509 | *PPP1R14C* | 8.72 | 8.45E-03 |
| ILMN_2687570 | *SRGAP2* | 8.56 | 5.93E-03 |
| ILMN_2539109 | *LOC383308* | 8.11 | 4.81E-02 |
| ILMN_2710159 | *MGC41689* | 7.80 | 2.98E-02 |
| ILMN_2607215 | *2310076L09RIK* | 7.77 | 2.01E-02 |
| ILMN_1217254 | *ATRNL1* | 7.52 | 2.78E-02 |
| ILMN_2665411 | *NNMT* | 7.42 | 2.68E-02 |
| ILMN_1242107 | *COX7A2L* | 7.02 | 4.01E-09 |
| ILMN_1217331 | *MCM6* | 6.61 | 4.94E-02 |
| ILMN_2546624 | *NUDEL-PENDING* | 6.47 | 6.00E-03 |
| ILMN_1221102 | *ARL5A* | 6.32 | 7.65E-03 |
| ILMN_1221939 | *LOC381556* | 6.14 | 6.00E-03 |
| ILMN_2764391 | *MUC6* | 6.10 | 4.54E-03 |
| ILMN_2680601 | *C130090K23RIK* | 6.00 | 2.48E-02 |
| ILMN_2746483 | *WDR82* | 5.75 | 3.71E-02 |
| ILMN_3128535 | *SAMD4* | 5.70 | 9.87E-05 |
| ILMN_1250126 | *C130032B16RIK* | 5.66 | 2.70E-03 |
| ILMN_2875251 | *ANG* | 5.55 | 7.57E-04 |
| ILMN_2946970 | *THEM5* | 5.41 | 7.45E-03 |
| ILMN_1225825 | *LOC100039175* | 5.27 | 3.90E-04 |
| ILMN_2774882 | *1110005F07RIK* | 5.14 | 2.79E-07 |
| ILMN_2620893 | *DLGAP2* | 4.99 | 4.46E-02 |
| ILMN_1215149 | *CRTC1* | 4.88 | 2.72E-02 |
| ILMN_1221006 | *LOC383370* | 4.76 | 1.30E-02 |
| ILMN_2747430 | *ATP7A* | 4.49 | 1.60E-04 |
| ILMN_2459211 | *DGKH* | 3.80 | 1.81E-02 |
| ILMN_2645460 | *2410146L05RIK* | 3.74 | 4.35E-02 |
| ILMN_1253773 | *NUDT6* | 3.70 | 1.95E-02 |
| ILMN_2637742 | *PADI1* | 3.67 | 5.80E-04 |
| ILMN_1225528 | *TRIB3* | 3.49 | 8.88E-03 |
| ILMN_2763772 | *RPAP3* | 3.39 | 4.07E-02 |
| ILMN_2864309 | *OTTMUSG00000000971* | 3.37 | 1.28E-02 |
| ILMN_3150536 | *1200015F23RIK* | 3.33 | 1.48E-05 |
| ILMN_2698334 | *4930486L24RIK* | 3.30 | 4.76E-02 |
| ILMN_2722769 | *TTPAL* | 3.30 | 6.76E-04 |
| ILMN_1231074 | *SP7* | 3.23 | 5.80E-04 |
| ILMN_2693858 | *D14ERTD449E* | 3.11 | 6.40E-06 |
| ILMN_1237999 | *9030016H15RIK* | 3.03 | 4.22E-02 |
| ILMN_1217098 | *DRD4* | 3.02 | 4.99E-02 |
| ILMN_2737296 | *LARS2* | 2.99 | 1.97E-03 |
| ILMN_1250852 | *CCDC5* | 2.95 | 3.96E-03 |
| ILMN_2747480 | *SRPR* | 2.78 | 1.17E-03 |
| ILMN_2700233 | *CCNG2* | 2.77 | 2.27E-03 |
| ILMN_2529392 | *LOC331139* | 2.77 | 2.78E-02 |
| ILMN_1219686 | *ESD* | 2.71 | 1.84E-03 |
| ILMN_2970834 | *PEX19* | 2.69 | 4.24E-02 |
| ILMN_2502542 | *UAP1* | 2.69 | 6.00E-03 |
| ILMN_1248465 | *ZCCHC3* | 2.66 | 2.11E-04 |
| ILMN_1230048 | *FXR2* | 2.61 | 1.17E-02 |
| ILMN_2588055 | *ACTB* | 2.53 | 4.55E-02 |
| ILMN_2437216 | *2610001E17RIK* | 2.52 | 1.28E-02 |
| ILMN_1246351 | *9130416B15* | 2.51 | 4.81E-02 |
| ILMN_2939277 | *SNCG* | 2.51 | 4.41E-02 |
| ILMN_2482572 | *FLNB* | 2.50 | 2.75E-05 |
| ILMN_1253848 | *NEO1* | 2.44 | 4.46E-02 |
| ILMN_1234842 | *MSI2H* | 2.40 | 2.40E-04 |
| ILMN_2613469 | *PSMB5* | 2.39 | 2.11E-06 |
| ILMN_2703563 | *STAC2* | 2.36 | 4.06E-02 |
| ILMN_1245850 | *4933427D14RIK* | 2.33 | 7.45E-03 |
| ILMN_2804523 | *DUSP7* | 2.29 | 3.75E-02 |
| ILMN_2862538 | *COL12A1* | 2.28 | 1.45E-02 |
| ILMN_2441501 | *CLSTN1* | 2.25 | 3.64E-03 |
| ILMN_2706101 | *SNX5* | 2.19 | 1.45E-02 |
| ILMN_2464201 | *3110021A11RIK* | 2.17 | 7.99E-03 |
| ILMN_2628567 | *PHLDA3* | 2.16 | 1.91E-02 |
| ILMN_2644504 | *RNASE4* | 2.14 | 3.57E-02 |
| ILMN_1220121 | *KIF23* | 2.14 | 2.30E-02 |
| ILMN_1232456 | *NNAT* | 2.14 | 2.56E-02 |
| ILMN_1255422 | *CCRN4L* | 2.12 | 4.53E-02 |
| ILMN_1235361 | *EG244911* | 2.06 | 4.23E-02 |
| ILMN_2770667 | *ACIN1* | 2.06 | 2.65E-02 |
| ILMN_1246770 | *YBX3* | 2.05 | 3.78E-04 |
| ILMN_1254634 | *ACPL2* | 2.04 | 1.14E-03 |
| ILMN_3132361 | *NCOA4* | 2.03 | 7.68E-03 |
| ILMN_2819558 | *BACH2* | 2.03 | 2.56E-02 |

| ILMN_1229454 | *CUEDC1* | -9.12 | 3.13E-02 |
| --- | --- | --- | --- |
| ILMN_1243252 | *SLC30A10* | -8.37 | 1.48E-02 |
| ILMN_2747634 | *GPR126* | -8.08 | 8.73E-03 |
| ILMN_2617149 | *F730015K02RIK* | -8.06 | 3.24E-05 |
| ILMN_2627377 | *4921525H12RIK* | -7.28 | 7.45E-03 |
| ILMN_2537111 | *LOC384118* | -7.18 | 1.32E-02 |
| ILMN_2840247 | *ZFP157* | -7.12 | 2.73E-02 |
| ILMN_1223594 | *OTTMUSG00000025408* | -7.02 | 1.28E-02 |
| ILMN_2681248 | *MYO3B* | -6.24 | 8.42E-04 |
| ILMN_1239900 | *LOC381333* | -6.20 | 4.21E-02 |
| ILMN_1236306 | *GM288* | -6.15 | 4.77E-02 |
| ILMN_2700364 | *2300005B03RIK* | -6.00 | 1.39E-02 |
| ILMN_1247327 | *ATXN7L3* | -5.83 | 2.30E-02 |
| ILMN_3004065 | *WBP2NL* | -5.66 | 2.68E-02 |
| ILMN_1229544 | *LOC100041569* | -5.31 | 7.23E-06 |
| ILMN_3095356 | *LOC545013* | -4.96 | 1.29E-02 |
| ILMN_2644531 | *ENAM* | -4.89 | 1.28E-02 |
| ILMN_2617639 | *9530053H22* | -4.77 | 5.03E-03 |
| ILMN_1227979 | *OLFR305* | -4.61 | 2.97E-02 |
| ILMN_3080159 | *PNPT1* | -4.59 | 1.95E-02 |
| ILMN_1215298 | *A930030D01RIK* | -4.39 | 3.81E-03 |
| ILMN_2619885 | *HELLS* | -4.13 | 4.42E-02 |
| ILMN_2705097 | *DEADC1* | -3.41 | 1.56E-02 |
| ILMN_1214706 | *C430014G13RIK* | -3.28 | 8.48E-04 |
| ILMN_2979639 | *H2-DMB2* | -2.79 | 1.28E-02 |
| ILMN_1246772 | *B430305P08RIK* | -2.79 | 2.60E-02 |
| ILMN_2534635 | *LOC272683* | -2.60 | 3.36E-02 |
| ILMN_2482821 | *LOC636875* | -2.54 | 3.96E-02 |
| ILMN_2788223 | *KNG1* | -2.53 | 1.66E-02 |
| ILMN_1219084 | *1700052K11RIK* | -2.47 | 8.81E-03 |
| ILMN_2984434 | *NLE1* | -2.44 | 1.97E-04 |
| ILMN_2925094 | *MPO* | -2.42 | 5.13E-03 |
| ILMN_2972521 | *AGTR1A* | -2.40 | 2.06E-02 |
| ILMN_2450155 | *IGLC2_J00595_IG_LAMBDA_CONSTANT_2_14* | -2.37 | 2.53E-02 |
| ILMN_2467429 | *D6MIT97* | -2.24 | 3.08E-02 |
| ILMN_2916571 | *IL3RA* | -2.21 | 4.04E-02 |
| ILMN_1229830 | *ZFHX1B* | -2.18 | 1.74E-03 |
| ILMN_2559669 | *PBX1* | -2.17 | 5.71E-03 |
| ILMN_1221817 | *CD74* | -2.14 | 1.45E-02 |
| ILMN_1241260 | *D330011G23RIK* | -2.11 | 4.69E-02 |
| ILMN_3089584 | *CD74* | -2.09 | 1.28E-02 |
| ILMN_1213090 | *2010305C02RIK* | -2.04 | 7.65E-03 |
| ILMN_2534921 | *LOC386486* | -2.02 | 1.76E-04 |

**Supplementary Table S2. Numbers and percentages of outer hair cell (OHC) loss observed in the homozygous p.V37I knock-in mice**

| **Type** | **Turns** | **Missing OHCs/ total OHCs** | | | |
| --- | --- | --- | --- | --- | --- |
|  |  | **Sample-1** | **Sample-2** | **Sample-3** | **percentages of OHC loss (%)** |
| **KI** | **Basal** | 1/79 | 1/76 | 1/82 | 1.3 |
|  | **Middle** | 3/75 | 3/79 | 3/80 | 3.8 |
|  | **Apical** | 3/76 | 3/78 | 3/81 | 3.8 |
| **WT** | **Basal** | 0/73 | 0/78 | 0/75 | 0 |
|  | **Middle** | 0/88 | 0/86 | 0/82 | 0 |
|  | **Apical** | 0/83 | 0/82 | 0/80 | 0 |

**Supplementary Figure S1. The schematic illustration of the knock-in strategy for p.V37I variant of mouse *Gjb2*.**


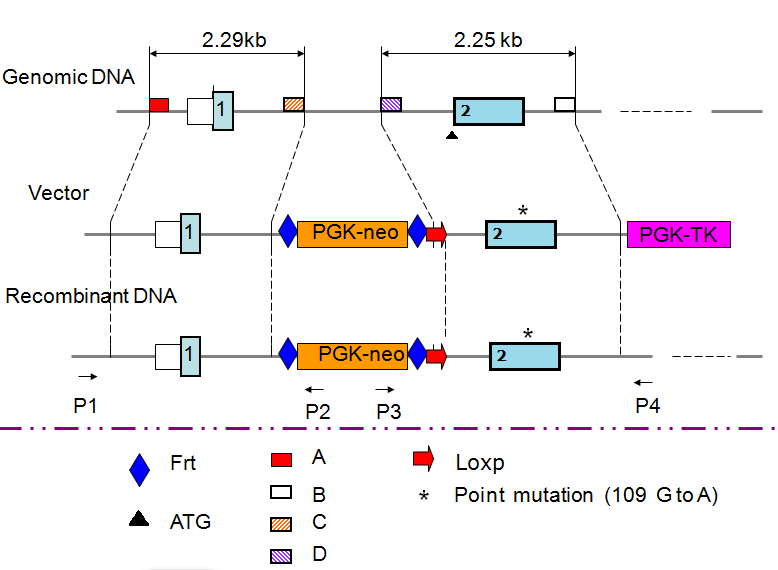

Supplement: Supplementary Information [file srep33279-s1.docx]
